# Particle and Chemical Emissions During Fused Filament Fabrication (FFF) Using Commercial ABS- and PET-G-Based Filaments

**DOI:** 10.3390/ma19091895

**Published:** 2026-05-04

**Authors:** Elżbieta Dobrzyńska, Tomasz Jankowski, Monika Borucka

**Affiliations:** Central Institute for Labour Protection—National Research Institute, Czerniakowska 16, 00-701 Warsaw, Poland

**Keywords:** fused filament fabrication, particles, volatile organic compounds, additives to filaments, phthalates, emission

## Abstract

**Highlights:**

- The ABS-based filaments tested in this study emitted noticeably higher concentrations of volatile organic compounds (VOCs) and ultrafine particles (55–90 nm) than the tested PET-G-based filaments.
- Emission profiles varied between the same filament type from different manufacturers, indicating formulation-dependent behavior
- The PET-G-based filaments tested in this study emitted higher levels of endocrine-disrupting phthalic acid esters (PAEs)
- The highest particle emission rates occurred during the initial warm-up and start of the printing process.

**Abstract:**

Despite its advantages, 3D printing may expose users to volatile organic compounds (VOCs) and particle emissions. Emissions from commercially available acrylonitrile-butadiene-styrene (ABS)- and polyethylene terephthalate glycol (PET-G)-based filaments were analyzed to evaluate differences among material formulations from multiple manufacturers. Chamber-based measurements and complementary thermal decomposition experiments were used to characterize particle number concentrations and chemical emissions. The highest particle emissions occurred during the initial warm-up and the final stages of the printing process. The ABS-based filaments tested in this study exhibited higher VOC emissions, dominated by styrene (up to 264.75 μg/m^3^), and particle number concentrations approximately one order of magnitude greater than those measured for the tested PET-G-based filaments. The dominant particle sizes ranged from 55 to 90 nm. PET-G-based filaments showed higher thermal stability but emitted notable concentrations of acetaldehyde (up to 70.93 μg/m^3^) and phthalic acid esters. Both filament types released compounds of potential health concern, including formaldehyde and reprotoxic substances such as dibutyl phthalate and bis(2-ethylhexyl) phthalate. Differences were observed among fibers made from the same polymer type, indicating the influence of formulation-specific factors. These results underscore the importance of material selection and adequate ventilation to minimize exposure during 3D printing.

## 1. Introduction

3D printing is widely used in industry and among hobbyists, due to its broad availability and variety of consumables. Recent advances in additive manufacturing combine high-performance materials with precision printing to meet strict industrial needs. In areas such as aerospace and biomedical implants, 3D printing streamlines manufacturing and enables the production of lightweight, high-performance structures. However, its health impacts and necessary safety measures remain poorly understood [1].

The most popular 3D-printing technology is FFF (Fused Filament Fabrication), in which the printer, guided by a computer-generated model, heats and melts thermoplastic filament, which is then extruded through a nozzle and deposited in successive layers to create a precise replica of the 3D model [2]. The range of filaments suitable for this technology has been extensively described in the relevant literature, and their selection depends primarily on the desired properties of the finished object. The most popular filament types are PLA, ABS, PET-G, TPU, ASA, and nylon. Meanwhile, the growing interest in this type of printing requires a critical analysis of the safety issues associated with different filaments.

During the printing process, filaments may release solid particles (ranging from 1 to 100 nanometers in diameter) and chemical compounds, the exact quantities and compositions of which are difficult to determine; prolonged inhalation of these particles and compounds may be harmful to health [3,4,5,6]. Understanding the emission process and exposure to emitted particles and chemical compounds during 3D printing is crucial for ensuring appropriate occupational safety and hygiene for workers and, consequently, for the development of safer 3D-printing technologies [7,8,9,10]. Excessive exposure to volatile organic compounds (VOCs) released during printing can lead to eye, nose, and throat irritation, headaches, or loss of coordination. A potential health concern is the impact of certain identified substances that may be carcinogenic, mutagenic, or reprotoxic [11]. Ultrafine particles (UFP) or nanoparticles generated during 3D printing are also associated with adverse health effects [9], raising concern about their inflammatory, pulmonary, and cardiovascular consequences. Particles smaller than 100 nm can enter the bloodstream and be transported to various internal organs, causing adverse health effects [12,13,14,15,16].

Compounds released during FFF printing result from the thermal breakdown of the base polymer or additives present in the printing material [17,18]. Different polymers can emit monomers and oligomers due to degradation and chemical changes at high temperatures [19]. Many 3D-printer filaments also contain unreacted initiators or additives that influence volatile organic compound emissions. Additives such as dyes, plasticizers, and stabilizers are mixed with filaments to enhance product performance [20,21], but they also increase uncertainty about exposure to particulates. Tedla and Rogers [22] observed that particle emissions can increase at higher temperatures, particularly with metal-filled filaments, potentially affecting human exposure.

The filament’s material, additives, brand, color, and batch all affect the amount and type of organic compounds emitted. Printer brand and printing settings—such as extrusion temperature, bed temperature, printing frequency, and nozzle wear—also matter [23,24,25].

The literature reports inconsistencies in the composition of the volatile mixture emitted during filament printing [23,26]. The lack of a uniform analysis methodology for emissions hinders comparisons across studies and process parameters, making it difficult to draw clear conclusions about their impacts. While emissions of volatile organic compounds and ultrafine particles during FFF printing have been repeatedly reported, there is limited comparative data on how specific manufacturer formulations affect the emission of toxic substances. Most research treats all filaments of the same type as identical, despite the addition of proprietary dyes, stabilizers, and chemicals, which can alter emission profiles and potentially change toxicity. There is insufficient information on the effects of these additives on filament emissions across brands. Additionally, the impact of processing temperature on emission profiles remains insufficiently explored. Although manufacturers provide recommended nozzle temperatures, users often adjust settings to optimize print quality or mechanical properties, which can lead to varying emission impacts. Because thermal degradation of polymers is strongly temperature-dependent, even minor deviations can increase the release of volatile organic compounds, ultrafine particles, and hazardous byproducts.

This publication aims to present and compare the safety aspects of using two of the most popular filament types, ABS and PET-G (considered a less harmful alternative in terms of chemical and particle emissions) for 3D printing. Polylactic acid (PLA) is one of the most commonly used filaments and is also considered a safer alternative. The literature indicates that PLA typically emits lower levels of volatile organic compounds (VOCs) than ABS, with lactide as the predominant emission product, along with carbonyl compounds such as acetaldehyde. Recent studies show that under certain printing conditions, it can still generate ultrafine particles and potentially irritating compounds. Nevertheless, we decided to limit our research to engineering-grade filaments (ABS and PET-G), which are more commonly used in professional and industrial applications and tend to emit higher levels of pollutants.

At the same time, the study aimed to determine the effect of additives to the base polymer on emissions of particles and volatile organic compounds, which are often manufacturers’ trade secrets. For this reason, the study focused on comparing filaments made from the same base polymers, sourced from three different manufacturers, with identical parameters or color, and used for printing under the same controlled conditions. Additionally, experiments were conducted under limited oxygen conditions, which are uncommon in standard 3D printing but possible during emergencies, such as poor ventilation, localized overheating, or disrupted airflow. This information is intended to raise awareness among 3D-printing users and filament manufacturers, and to promote the safe use of 3D-printing technology in a variety of settings, from home workshops to professional applications.

## 2. Materials and Methods

### 2.1. Test Materials

Six different materials were used for the study: three filaments labeled ABS 1–3 (acrylonitrile butadiene styrene copolymer) and three filaments labeled PET-G 1–3 (glycol-modified polyethylene terephthalate), all in one color (white) with a diameter of 1.75 mm, and otherwise the same parameters from 3 different manufacturers (Table 1). ABS 1, 2, and 3 (and PET-G 1–3) refer to three different commercial formulations; we produced three replicates of each material (n = 3 per filament).

It should be noted that the as-received filaments were not subjected to prior physicochemical characterization. Therefore, the detailed composition, thermal stability, and specific additive content of each material were not independently verified before emission testing.

### 2.2. Steady-State Tube Furnace Experiments

To identify selected chemical substances that may be released during 3D printing, we used an experimental setup. This consisted of a steady-state tube furnace and a Fourier transform infrared spectroscopy (FT-IR) with a Gasmet DX4000 portable analyzer (Gasmet Technologies Oy, Vantaa, Finland). The steady-state tube furnace (Purser furnace, ISO 19700 [27]) was designed to reproduce real fire conditions under controlled oxygen-deficient atmospheres. This enabled the generation of toxic combustion products in accordance with ISO 19706 [28]. In this study, the furnace simulated the operating temperatures of selected 3D-printing filaments. At the target temperatures, we determined the concentrations of carbon dioxide (CO_2_), carbon monoxide (CO), hydrogen cyanide (HCN), hydrogen chloride (HCl), nitrogen dioxide (NO_2_), ammonia (NH_3_), and light hydrocarbons. The measured hydrocarbons included methane (CH_4_), ethane (C_2_H_6_), propane (C_3_H_8_), ethylene (C_2_H_4_), and hexane (C_6_H_14_).

Additionally, to identify volatile and semi-volatile compounds formed during the test, the gas samples were collected using a carboxen/polydimethylsiloxane (CAR/PDMS) solid-phase microextraction (SPME) fiber (Supelco, Bellefonte, PA, USA), in accordance with ISO 19701 [29]. Before sampling, the filament was conditioned in the GC injection port as recommended by the manufacturer. The SPME device was then exposed to the effluent in the mixing chamber for 15 min, followed by immediate thermal desorption in the GC injector. Analyses were performed using a gas chromatograph (GC 7890A, Agilent Technologies, Santa Clara, CA, USA) coupled with a mass spectrometer (MSD 5975, Agilent Technologies, USA). The limit of quantification (LOQ) was defined as a signal-to-noise ratio of approximately 10:1. Chromatographic peaks were identified by comparing acquired mass spectra with the NIST MS Library, applying a confidence (match) threshold of >90%.

### 2.3. Chamber Emission Testing

**Figure 1:**
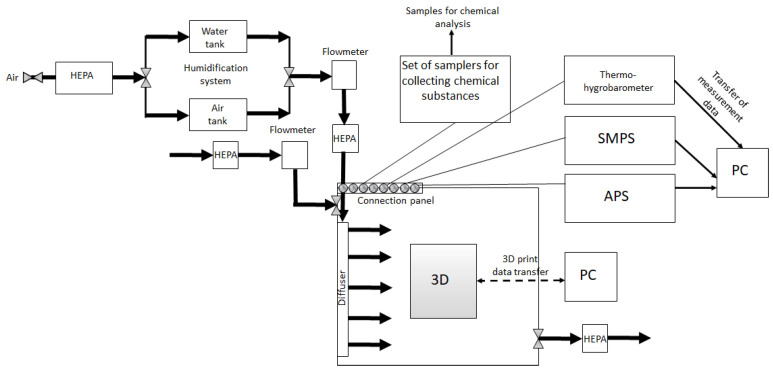
Schematic presentation of the testing station.

Emissions of VOCs and particles were measured during 3D printing in a stainless-steel chamber (100 × 100 × 77 cm) equipped with a sample-handling module. Tests ran in dynamic mode with air exchange, and temperature and humidity were controlled (Figure 1). Air temperature, humidity, and atmospheric pressure in the chamber were tracked with a LAB-EL LB-706B thermohygrobarometer (LAB-EL, Reguly, Poland). The HygroPalm HP23-A (Rotronic AG, Bassersdorf, Switzerland) monitored temperature and humidity in the humidification system.

An original Prusa i3 MK3 printer (Prusa Research a.s., Prague, The Czech Republic) with dedicated control software was used to print the standard cube object. Three ABS filaments (ABS 1–3) and three PET-G filaments (PET-G 1–3) from four different manufacturers were tested. The tests followed the ANSI/CAN/UL 2904 standard [30]. During 3D printing with individual filaments, the printer’s operating parameters remained constant. The extruder temperature was set to 255 °C for ABS and 245 °C for PET-G filaments. Air temperature inside the chamber was maintained within ±1 °C of the temperature after the extruder had warmed up. Relative humidity remained within 45% to 55%. The chamber, with a volume of less than 5 m^3^, had an air exchange rate between 1–5 h^−1^ ± 5%. During printer operation, airflow was directed at a linear velocity of 0.1–0.3 m/s.

Operating parameters of the printer used during 3D printing with specific filaments were as follows:

Extruder temperature: 255 °C (for ABS filaments) and 245 °C for PET-G filaments

Bed temperature: First layer: 100 °C; Subsequent layers: 110 °C

Extruder nozzle diameter: 0.4 mm

Print speed: Outer contours: 25 mm/s/Inner contours: 45 mm/s

Fill: 80 mm/s

Layer height: 0.15 mm

Fill: 70%, grid

During the test, the same 3D object was printed—a test cube, whose mass should be between 45.0 g and 46.0 g.

### 2.4. Chemical Analysis Methods

Combined samplers consisted of a 25 mm glass fiber filter (Waters, Milford, MA, USA) and a PUF foam cartridge, connected to an absorption tube with Orbo 43-treated Amberlite^®^ XAD^®^-2 (Supelco, Bellefonte, PA, USA) for PAE sampling. Sorption tubes containing 0.7–1.0 mm of activated carbon (ZUP “Analityk”) collected volatile organic compounds. Carbonyl compounds were collected using adsorption tubes with silica gel coated with 2,4-dinitrophenylhydrazine (Supelco). Aspirators sampled air at constant flow (e.g., AirCheck 2000 [SKC Inc., Eighty Four, PA, USA], Gilian LFS-113DC [Sensidyne, St. Petersburg, FL, USA], GilAir Plus [Sensidyne]). Certified phthalate, VOC, and carbonyl standards (Sigma-Aldrich, Steinheim, Germany) and solvents—acetonitrile (Merck, Darmstadt, Germany), dichloromethane (Sigma-Aldrich), acetone (J.T. Baker, Radnor, PA, USA), carbon disulfide (Honeywell, Charlotte, NC, USA), and MilliQ water—were used. A Hewlett-Packard gas chromatograph with a mass spectrometry detector and an RTX-5 silMS column (Restek, Bellefonte, PA, USA) analyzed VOCs and PAEs. An Elite LaChrom liquid chromatograph with a DAD detector and an Ultra C18 column (Restek) (Merck-Hitachi, Tokyo, Japan) analyzed carbonyls.

### 2.5. Particle Measurement Methods

The emitted particles were collected isokinetically directly into particle counters. The SMPS Model 3938 (TSI Inc., Shoreview, MN, USA) particle size analysis system and the APS Model 3321 (TSI Inc.) aerodynamic particle counter were used to determine the particle number concentration and size distribution emitted during 3D printing. The SMPS system consists of a Model 3776 CPC condensation particle counter (TSI Inc.) and a Model 3082 DMA electrostatic classifier (TSI Inc.). The measurement results were determined in two particle size ranges:

− particles in the range from 15 nm to 0.5 μm (SMPS measurement),
− particles in the range from 0.5 μm to 20 μm (APS measurement).

### 2.6. Data Analysis and Calculations

Particle emission rates (*PER*(*t*) as a function of time are calculated using *C_p_* based on a mass balance and are given by

(1) $$PER\left(t\right)=V(\frac{C_{p}\left(t\right)-C_{p}\left(t-∆t\right)exp(-\beta \cdot ∆t)}{∆t\cdot exp(-\beta \cdot ∆t)})$$

where

*β* [s^−1^] is the particle loss coefficient.

*C_p_* [cm^−3^] is the particle concentration.

Δ*t* [s] is the time interval between two successive data points.

*V* [m^3^]—chamber volume.

Total particle emission (*TP*) over the complete print job is the integral of *PER*(*t*) over the emission phase, and was calculated using the following formula:

(2) $$TP=\int_{t_{start}}^{t_{stop}}PER(t)$$

(3) $$dt=\sum_{t_{start}}^{t_{stop}}PER(t)\cdot ∆t$$

where

*t_start_* is the time when extrusion begins, or *C_p_* begins to increase

*t_stop_* is defined as follows:

If *t*_1%*PERmax*_ ≤ *t_print end_*

*t_stop_* _=_ *t_print end_*

else

*t_stop_* = *t*_1%*PERmax*_

where

t_1%PERmax_ is the time when *PER*(*t*) remains steady.

The t_print end_ is the time when the extrusion ends.

The emission rate (*ER*), expressed as micrograms per hour (µg/h), was calculated using the following formula:

(4) $${ER}_{g,i}=\;\frac{Q\;\left(C_{it}-C_{i0}\cdot exp\left(-\frac{Q}{V}\;\left(t-\;t_{0}\right)\right)\right)}{1-\exp\left(-\frac{Q}{V}\;\left(t-\;t_{0}\right)\right)}$$

where *ER_g_*_,*i*_ is the emission rate (µg/h) of a given compound.

*Q* [m^3^/h] is the inlet flow rate—air entering the chamber.

*C_it_* [µg/m^3^] is the concentration of the compound in the chamber.

*C_o_* [µg/m^3^] is the background concentration for a given chemical compound.

*t* [h] is the sampling time.

*V* [m^3^] is the chamber volume.

Based on the emission rate (ER), the emission yield [μg/g] is calculated as follows:

(5) $$\mathrm{Emission}\;\mathrm{yield}\;{Yield}_{g,i}=\;\frac{{ER}_{g,i}\cdot t_{print}}{m_{printed}}$$

where *m_printed_* is the printed object’s mass [g] and *t_printed_* is the printing time [h].

Emission rates (ER) and yields (Y) were calculated independently for each replicate (n = 3), and results are presented as mean values with standard deviations.

## 3. Results and Discussion

### 3.1. Emission Studies in the Chamber

Measurements taken in a test chamber during printing with each tested filament confirmed the presence of a complex mixture of volatile organic compounds (VOCs), including carbonyl compounds and phthalic acid esters. Notably, the tested filaments varied in the emission levels of individual chemical compounds. In particular, attention was paid to substances hazardous to printer operators’ health, such as formaldehyde, acetaldehyde, styrene, dibutyl phthalate, and bis(2-ethylhexyl) phthalate.

**Figure 2:**
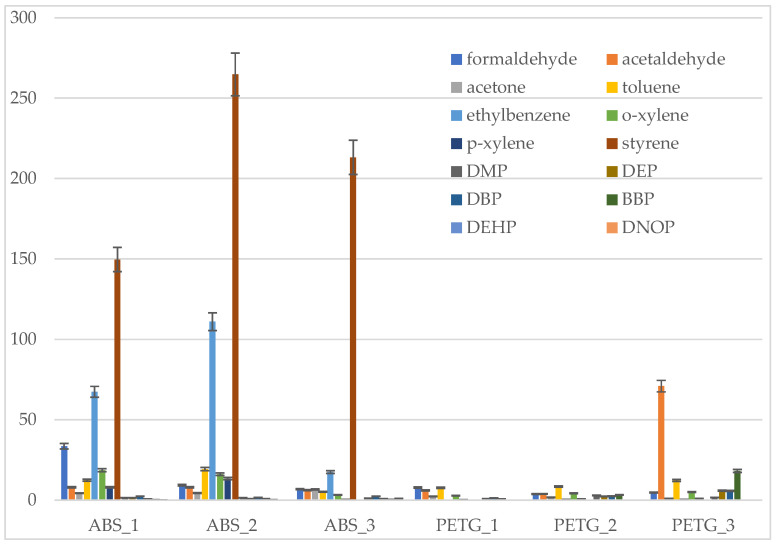
Comparison of individual VOC emissions during printing with ABS and PET-G filaments.

Depending on the filament tested, the following compounds were identified: xylenes, ethylbenzene, styrene, cumene (isopropylbenzene), isobutyl acrylate, 2,4-dimethylhexane, toluene, and siloxanes. The dominant chemical compound emitted during 3D printing with ABS filaments was styrene, the main component of ABS filaments. It is harmful if inhaled, and can cause eye and skin irritation. However, noticeable differences in the concentration of this compound were observed among the tested ABS filaments 1–3, sourced from different manufacturers (Figure 2). The highest concentration, 264.75 µg/m^3^, was measured in the ABS 2 filament. Notable differences were observed in the emission levels of individual VOCs for both ABS and PET-G filaments, as well as between filaments of the same type (Figure 2). Printing with PET-G filaments resulted in lower emissions of VOCs (at the levels of 1.13–12.29 µg/m^3^) than with ABS filaments. The highest concentrations of toluene were measured at 7.68 µg/m^3^ (PET-G 1) and 12.29 µg/m^3^ (PET-G 3).

Carbonyl compounds released during printing with ABS filaments were measured at the levels of 1.49–33.56 µg/m^3^, and for PET-G (1.17–32.62) µg/m^3^. The average formaldehyde concentrations ranged from (6.85–33.56) µg/m^3^ for three ABS filaments and (3.82 to 7.92) µg/m^3^ for PET-G 1–3 filaments. The highest formaldehyde concentration (33.56 µg/m^3^) was measured during 3D printing with ABS-1 filament. It should be emphasized that formaldehyde may be a potential decomposition product of a polymer. According to the CLP Regulation [31], it is also a compound that irritates the upper respiratory tract and skin, and is classified as a category 1B carcinogen for humans and a category 2 mutagen. Acetone concentrations ranged from (4.37–6.69) µg/m^3^ for ABS and (1.12–2.91) µg/m^3^ for PET-G. Acetaldehyde concentrations ranged from (6.18 to 8.13) µg/m^3^ for ABS, and from (3.93–70.93) µg/m^3^ for PET-G filaments. Between the filaments of the same type, the differences in the highest acetaldehyde concentrations measured during 3D printing was with PET-G-3 filament (70.90 µg/m^3^). Acetaldehyde is classified as a category 1B carcinogen to humans and a category 2 mutagen [31].

Phthalic acid esters were tested both in the inhalable (i.e., the fraction of aerosol that enters through the nose and mouth and poses a health risk after deposition in the respiratory tract) and in the gas fraction. The sum of PAEs across both fractions shows slight differences among the three ABS filaments tested, but much greater differences with the PET-G filaments. Higher concentrations of phthalic acid esters released from the PET-G filaments result from their chemical composition, i.e., a filament based on glycol-modified polyethylene terephthalate.

**Figure 3:**
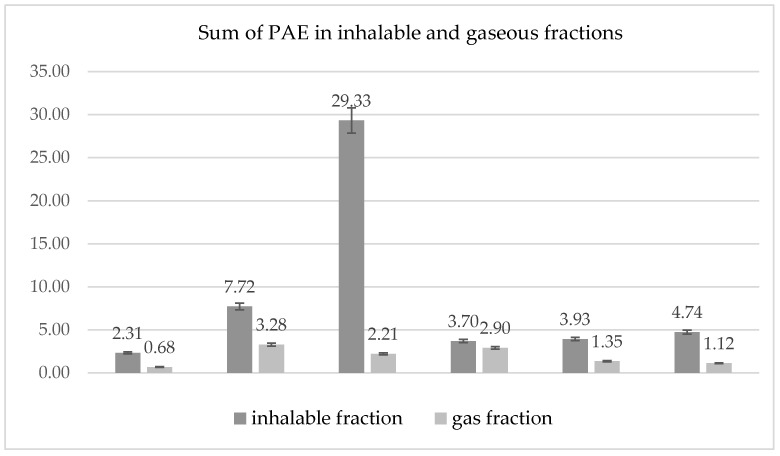
Total PAEs in the inhalable and gaseous fractions for ABS and PET-G filaments.

The highest total PAE concentration in the inhalable fraction measured during printing with ABS filaments was 4.74 µg/m^3^ (ABS 3), while in the gas phase it was 2.9 µg/m^3^ (ABS 1). For PET-G filaments, the sum of PAEs in the inhalable fraction ranged from 2.31 (PET-G 2) to 29.33 µg/m^3^ (PET-G 3), depending on the filament manufacturer. A comparison of the total PAE results for both fractions (inhalable and gaseous) shows slight differences among the three ABS- and PET-G-based filaments (Figure 3).

**Figure 4:**
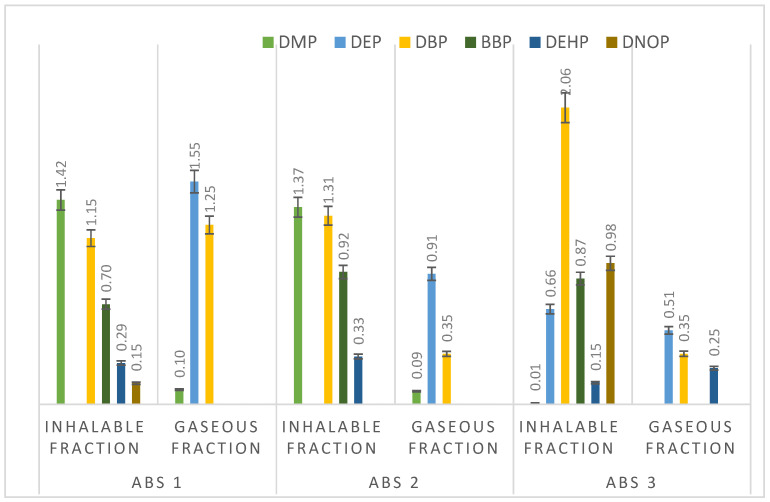
Distribution of individual PAEs emitted during printing with ABS filament.

The presence of PAEs, which disrupt the endocrine system, requires action. In particular, the detection of phthalates in both gaseous and inhalable fractions raises concerns regarding long-term exposure. Printer users must implement appropriate preventive and protective measures. It should be noted, however, that this study does not constitute a formal risk assessment. In the inhalation fraction, the main compounds identified were dimethyl phthalate (DMP), benzyl butyl phthalate (BBP), dibutyl phthalate (DBP), and bis(2-ethylhexyl) phthalate (DEHP). Diethyl phthalate (DEP) appeared only in the ABS3 application. Di-n-octyl phthalate (DNOP) appeared only in ABS1 and ABS3. DMP was the dominant phthalate in all air samples taken during printing with ABS filaments. The exception was ABS-3, where DBP was highest. In the gaseous phase, DMP, DEP, and DBP were detected. DEP was the dominant species. Its concentrations were 0.51, 0.91, and 0.55 µg/m^3^ in air samples analyzed during printing with ABS 1–3 filaments, respectively (Figure 4).

When printing with PET-G filament, the dominant compound in the inhalable fraction was BBP, except for PET-G 1 filament, where a higher concentration of DBP was measured. DEHP and DNOP were not detected in PET-G samples, indicating variability in phthalate composition depending on filament formulation, and that the phthalate profile differs from that of ABS and is dominated by other compounds such as BBP and DBP.

Phthalate compounds exhibit endocrine-disrupting properties (EDC). DBP, BBP, and DEHP are classified as Category 1 endocrine disruptors, indicating that there is sufficient evidence that their adverse effects on human health or wildlife are hormone-related, or that reliable test results indicate that the observed effects are hormone-related. DBP, BBP, and DEHP are Category 1B reproductive toxicants (Repr 1B) with the phrase H360-Df: May damage the unborn child. They are suspected of damaging fertility. The adverse effects of these substances may include the following: fertility disorders, sexual organ development disorders, hormone-dependent cancers (including breast, prostate, ovarian, and testicular cancer), fetal damage, including damage to the nervous system, as well as metabolic disorders, obesity, and diabetes.

It should be noted that the emissions observed in this study correspond to the recommended printing temperatures; however, polymer degradation mechanisms are highly temperature-sensitive. An increase in extrusion temperature typically intensifies chain-breaking reactions, accelerates oxidation processes, and promotes the release of monomers, oligomers, and additives. Higher temperatures may increase styrene formation from ABS or increase the emission of aldehydes and phthalates from PET-G due to the decomposition of additives and plasticizers. Therefore, the emission levels reported in this publication likely reflect baseline conditions, whereas higher temperatures may increase emissions.

For each group of compounds identified during printing with these filament types, emission rates (ER) and emission yields (Y) were calculated in accordance with ANSI/CAN/UL 2904. The results for three different ABS-based filaments are presented in Table 2, and for PET-G-based filaments are shown in Table 3.

The ER values for ABS1 ranged from 0.21 µg/h (DNOP) to 210.97 µg/h (styrene), for ABS2 from 0.47 µg/h (DEHP) to 373.3 µg/h (styrene), and for ABS3 from 0.57 µg/h (DEHP) to 300.55 µg/h (styrene).

The ER values for PET-G1 ranged from 0.06 µg/h (DMP) to 11.2 µg/h (formaldehyde); for PET-G2, they were below 0.1 µg/h for each chemical compound; and for PET-G3, they ranged from 1.4 µg/h for m- and p-xylenes to 100.01 µg/h for acetaldehyde. Each filament behaves reproducibly, and differences among them exceed experimental variability. Relative standard deviations were generally below 10%.

According to the measurements, none of the tested filaments had ER emission factor values for individual chemical compounds that exceeded the permissible value specified in ANSI/CAN/UL 2904 [30]. Although none of the tested filaments exceeded specific emission rate limits under the studied conditions, the detection of Category 1B carcinogens (substances suspected of causing cancer in humans, such as formaldehyde and acetaldehyde) and Group 1 endocrine-disrupting chemicals (dibutyl phthalate, and di(2-ethylhexyl) phthalate) supports adopting a precautionary approach. These compounds indicate a potential health risk, especially with long-term or repeated exposure. The presence of substances with established occupational exposure limits (OELs) highlights the need for operator awareness, documented risk assessment, and appropriate protective measures. However, the concentrations measured in this study did not exceed permissible limits, which suggests that, under controlled conditions, the immediate risk may be limited.

It should be emphasized that the present study focuses on identifying and quantifying emitted chemical compounds and particles during 3D printing and does not constitute a formal risk assessment. Although hazardous substances, such as formaldehyde, acetaldehyde, and phthalates were detected, the actual health risk depends on multiple factors, including exposure duration, concentration, ventilation conditions, and user behavior. A comprehensive risk assessment would require integration of these parameters with established exposure limits.

### 3.2. Particle Emission

Studies on particle emissions from 3D printers have determined the average particle size distribution, changes in average particle number and mass concentrations, and the average geometric diameter during printing.

**Figure 5:**
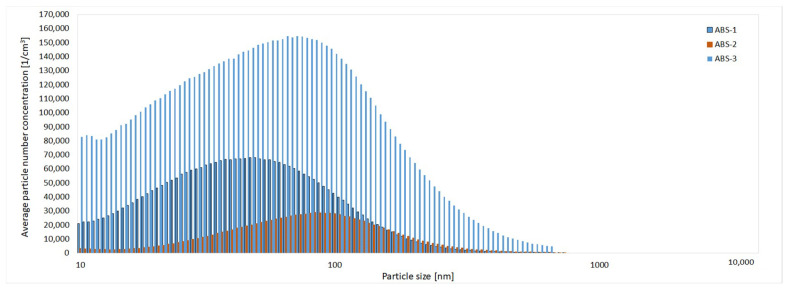
Average particle size distribution in the 3D-printing process of ABS filaments.

For all filaments, particles larger than 0.5 μm were negligible. Dominant particle sizes were as follows: ABS-1 (55 nm), other ABS (75 nm, 90 nm), PET-G 1 and 2 (65 nm), and PET-G 3 (80 nm). Figure 5 illustrates particle size distribution changes during ABS 3D printing. 

**Figure 6:**
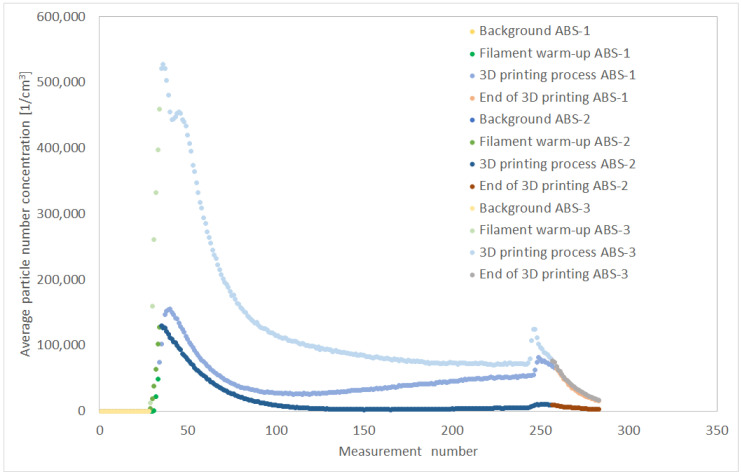
Changes in the average particle number concentration from 15 nm to 0.5 μm during 3D printing with ABS-based filament from different manufacturers.

Building on the previous discussion, the relationships between the instantaneous number concentration [particles/cm^3^] during the measurement enabled characterization of the individual stages of 3D printing (Figure 6).

For each filament considered, the highest particle concentration occurred at the start of the 3D-printing process. After an initial temporary increase, the concentration decreased during printing and then stabilized at a level several times lower. At the end of the 3D-printing process, another temporary increase in concentration occurred, followed by a rapid drop. The highest emission rates observed during the initial warm-up phase and at the end of the printing process indicate that effective mechanical ventilation is particularly important at these stages.

The total particle number concentration over the entire measurement range (15 nm to 20 μm) was obtained by integrating the particle number concentrations measured by the SMPS and APS instruments. To characterize the amount of particle emissions from the 3D printer, the following relationships were determined:

- changes in particle number concentration over time, *C_p_*(*t*), during the 3D printing process
- changes in the particle emission rate, *PER*(*t*), during the 3D printing process were also analyzed to further characterize these emission patterns.

The highest *PER*(*t*) particle emission rate occurred during the filament-heating stage and at the beginning of 3D printing. For example, with ABS-3 filament, PER(t) increased from 5.52 × 10^−2^ [s^−1^] to 2.01 × 10^3^ [s^−1^], before stabilizing at 6.24 × 10^1^ [s^−1^]. At the end of the 3D-printing stage, a rapid but temporary increase in *PER*(*t*) was observed for the ABS-3 filament, reaching 4.38 × 10^2^ [s^−1^].

The determined total particle number concentration, *C_p_*(*t*), was used for further calculations. To compare the emission levels from individual filaments during 3D printing, the following parameters were defined:

- total particle emission TP [—]
- particle yield Yield [g^−1^]
- average particle emission rate ERp [h^−1^].

**Figure 7:**
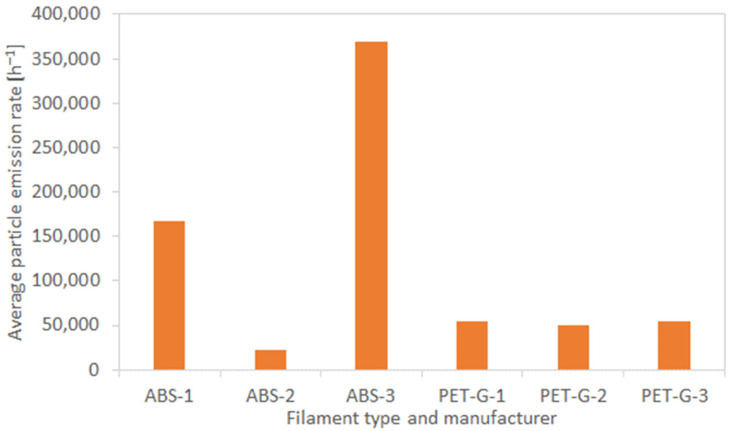
Comparison of the average particulate matter emission rate during 3D printing with ABS and PET-G-based filaments from different manufacturers.

Table 4 and Figure 7 compare particle-emission parameters during 3D printing using ABS and PET-G filaments from three manufacturers. For each measurement, the average particle size distribution in the chamber was determined. The graphs show the relationship between the average solid-particle emission rate [h^−1^] and filament type and manufacturer.

The number of particles released during 3D printing with ABS-2 filament is about ten times lower than with ABS-1 and ABS-3. Total particle emission (TP) for ABS-1 and ABS-3 was 5.00 × 10^5^ and 1.11 × 10^6^, while for ABS-2 it was 6.94 × 10^4^. Although the specific composition of ABS-2 is proprietary and was not independently characterized in this study, differences in particle emission among filaments based on the same base polymer (ABS) likely stem from compositional variations, including the types and concentrations of additives such as stabilizers, plasticizers, residual monomers, and pigments. These factors may influence thermal decomposition and particle formation during printing. This observation highlights the role of filament composition in emission behavior, but further research is needed to elucidate the exact mechanisms. These results show that both the polymer’s chemistry and the manufacturer’s formulation influence emissions.

The particle emission values during 3D printing with PET-G filament from different brands are similar. For example, the average emission rate (ERp) was 5.39 × 10^4^ [h^−1^] for PET-G-1 and 5.42 × 10^4^ [h^−1^] for PET-G-3.

Particle emission during 3D printing within this study with PET-G-based filament is about 10 times lower than with ABS-based filament. For example, the total TP for ABS-3 was 1.11 × 10^6^, while for PET-G-3 it was 1.63 × 10^5^.

Nanoparticle emissions from some filaments noticeably exceed the reference levels recommended by the IFA (40,000 nanoparticles/cm^3^) and the Instituto Nacional de Seguridad e Higiene en el Trabajo (INSST) in their guide to nanomaterials. Considering the WHO-recommended values (10,000 nanoparticles/cm^3^), it becomes clear that virtually all filaments tested in our study have the potential to exceed these limits [16]. Even low-emission filaments can reach dangerous levels under certain conditions, such as inadequate ventilation or incorrect temperature settings. Particle concentrations, though, may exceed WHO limits. This is more likely in poorly ventilated areas or during specific printing phases, such as warm-up and cool-down.

ABS-generated particle emission rates are approximately one order of magnitude higher than those of PET-G, suggesting that printing with ABS requires a greater air exchange rate compared to PET-G. Users should ensure adequate ventilation when printing with ABS. The specific air exchange rate required may vary depending on the printer type, room size, and other environmental factors, and our study does not provide a validated quantitative ventilation guideline. The user’s risk depends largely on environmental and operational conditions. To prevent exposure, 3D-printer users should work in well-ventilated rooms. They should consider enclosing the printer, minimizing time spent near operating printers—especially during the initial and final phases—and using personal protective equipment when ventilation is insufficient. If air exchange in a 3D-printing room is insufficient, effective mechanical ventilation is necessary. Mechanical ventilation can involve air exchange throughout the entire room or controlling the emission of particulate matter and chemicals from point sources, such as 3D printing. The most effective solution to prevent chemicals and particulate matter from entering the breathing zone and other rooms is to tightly enclose the space where the emissions originate during 3D printing. Many modern 3D printer models are equipped with enclosures featuring local mechanical ventilation. This enclosure minimizes the spread of particulate matter and chemicals into the room. It is recommended to use a closed chamber for the printer, equipped with a ventilation and air filtration system. Complete enclosures should be designed when constructing a 3D-printing workstation. When designing an enclosure for an existing 3D-printing workstation, it is essential to consider ergonomics and parameters that affect chemical and particulate matter emissions. Completely or partially enclosing a 3D-printer workstation is not always possible. Local exhaust systems equipped with suction nozzles and hoods (stationary or mobile) are used, connected to an air purification system. To ensure the safety of the 3D printer operator, proper settings for local exhaust parameters are paramount. A key element of air purification during 3D printing is the use of multi-stage air filtration systems. The configuration of air filtration systems depends on the required level of air cleanliness in the room. Therefore, the most commonly used filters are ISO ePM2.5 and ISO ePM1, high-efficiency filters (EPA, HEPA, ULPA), and activated carbon filters used for gas and odor adsorption.

### 3.3. Emission Studies in the Steady State Tube Furnace

To complement the study, materials were heated in a stationary tube furnace at the recommended operating temperature. It should be noted that this setup does not replicate the dynamic thermal and flow conditions (e.g., shear, residence time, and temperature gradients) present in a 3D printer nozzle during FFF. Therefore, these experiments are intended solely as complementary thermal degradation tests under controlled conditions.

Table 5 presents the average emission efficiency of the gaseous products released by the filaments at selected operating temperatures for 60 min. In the ABS-based filament group, clear differences in emission behavior were observed among the tested samples. The ABS-1 filament exhibited the highest overall emissions of several critical compounds, including CO_2_, NO_2_, HCl, and HCN, indicating that it undergoes more intensive thermal decomposition than the other ABS samples. ABS-3 showed comparatively low emissions for most compounds. The relatively high HCl emissions observed in ABS-1 may indicate that a chlorine-based flame retardant was incorporated during its production, contributing to the formation of hydrogen chloride gas upon heating. However, overall HCl emission levels remained relatively low.

The PET-G filament group exhibited a distinct emission profile and generally released more gas than the ABS group. PET-G-3 produced the highest NO_2_ emissions among all tested filaments, indicating more pronounced oxidative degradation. PET-G-2 showed the highest emissions of HCN and HCl, indicating the potential influence of additives that promote the release of nitrogen- and chlorine-containing compounds during heating. PET-G-3 also demonstrated the greatest overall CO_2_, emphasizing that carbon-based decomposition is particularly intense for this sample. In contrast, ammonia emissions from PET-G-1 and PET-G-3 were negligible, indicating that nitrogen-containing volatiles vary significantly between PET-G samples depending on their formulation.

Table 6, in turn, summarizes the average emission efficiency of light hydrocarbons emitted from ABS and PET-G filaments at the recommended operating temperatures.

In the ABS-based filaments group, ABS-3 emitted the lowest levels of nearly all measured hydrocarbons. ABS-1 and ABS-2 showed elevated emissions of C_2_–C_3_ hydrocarbons, particularly propane and ethane. This elevation likely results from more extensive thermal decomposition of the polymer chains in these samples, and possibly from the presence of more volatile additives or residual monomers.

A similar pattern was observed in the PET-G filaments. PET-G-1 and PET-G-3 generally released very low hydrocarbon levels, whereas PET-G-2 emitted noticeably higher amounts of methane, ethane, ethylene, and hexane. These elevated emissions in PET-G-2 may be attributed to differences in filament formulation, such as the presence of plasticizers or other additives that decompose more readily at higher temperatures, producing volatile hydrocarbons.

Table 7 presents a summary of the main chemical substances detected in the gas samples emitted by the tested filaments using the SPME-GC-MS method. In the samples collected during the heating of ABS-based filaments, a larger number of compounds was detected compared to the heating of PET-G-based filaments. Among the identified substances were compounds, such as acetaldehyde, acetophenone, benzaldehyde, styrene, toluene, and xylene, which have also been detected by other researchers [11]. The smallest number of different chemical substances was detected during the heating of the ABS 1 and PET-G 1 filaments.

The lower emission of volatile organic compounds from PET-G-based filaments is related to the polymer’s higher thermal stability. Thermal analysis indicates no notable VOC release between 220 and 270 °C, while major weight loss of about 90% occurs only above 330 °C [12].

Differences in emission behavior are largely related to thermal degradation mechanisms. PET-G undergoes limited degradation dominated by mild chain scission (breakage of polymer chains) and surface oxidation, producing mainly simple oxidation products such as carbon oxides. ABS (acrylonitrile butadiene styrene), composed of styrene, acrylonitrile, and butadiene, is more susceptible to thermal degradation, resulting in a broader spectrum of volatile organic compounds (VOCs), including styrene, toluene, xylene, benzaldehyde, and acetophenone. Consequently, ABS emissions are generally more chemically diverse. The presented studies were conducted at the processing temperatures recommended by the manufacturers. However, emission profiles are highly temperature-dependent, and higher temperatures are likely to increase the emissions of volatile organic compounds, ultrafine particles, and toxic degradation products. Further research should investigate the relationship between extrusion temperature and emission characteristics to support the development of more precise safety guidelines for 3D-printing processes.

In addition, further research is needed to better understand variability in emissions across material formulations, temperature settings, and printer configurations. Incorporating studies of exposure duration and the independent characterization of filament compositions could help clarify causal relationships between material properties and emission behavior. One limitation of the present study is the lack of independent raw material characterization before testing. Future studies should include analyses such as thermogravimetric analysis (TGA) or differential scanning calorimetry (DSC) to link composition and emissions, thereby strengthening causal conclusions.

## 4. Conclusions

The study demonstrates that commercially available 3D-printing filaments exhibit different volatile organic compound and particle emission profiles. Under controlled processing conditions, ABS-based filaments showed higher VOC emissions than PET-G-based filaments, with styrene as the dominant compound observed in ABS.

PET-G-based filaments generally exhibited lower total VOC emissions but showed relatively higher emissions of acetaldehyde and phthalic acid esters in some samples. Filaments of the same polymer type sourced from different manufacturers also showed noticeable differences in VOCs and particle emissions, suggesting that emission behavior may be linked to differences in material formulations. However, these observations should be interpreted with caution. Because the tested materials were not independently physicochemically characterized, it is not possible to directly attribute observed emission differences to specific chemical composition or additives. The results, therefore, reflect the overall emission behavior of commercial formulations rather than intrinsic polymer properties.

Emission patterns are consistent with the known literature in terms of the thermal degradation behavior of the tested polymers. ABS is associated with more extensive decomposition and a broader range of aromatic VOCs. At the same time, consistent with previously reported trends, PET-G exhibits greater thermal stability but may still generate aldehydes and phthalates under processing conditions.

Ultrafine particle emissions (55–90 nm) were highest during the initial warm-up and final printing stages. They were approximately one order of magnitude higher for ABS than for PET-G, indicating differences in measured particle concentrations under identical test conditions and process phases.

Although no regulatory emission limits were exceeded under the studied conditions, the detection of hazardous compounds, including formaldehyde, acetaldehyde, and endocrine-disrupting phthalates (DBP, DEHP, BBP), indicates potential occupational exposure concerns. This study does not constitute a formal risk assessment, as exposure conditions such as ventilation, duration, and user behaviors were not fully addressed. The study is also limited by the absence of a comprehensive statistical analysis, which precludes a thorough assessment of variability and inferential comparisons. Future studies should incorporate statistical analyses and physicochemical characterization techniques to better understand the relationships among material properties, processing conditions, and emission behaviors.

## Figures and Tables

**Table 1:** Filaments tested.

	Filament	Manufacturer	Color	Diameter [mm]
ABS 1	acrylonitrile butadiene styrene copolymer	Manufacturer A	White	1.75
ABS 2		Manufacturer B	White	1.75
ABS 3		Manufacturer C	White	1.75
PET-G 1	glycol-modified polyethylene terephthalate	Manufacturer A	White	1.75
PET-G 2		Manufacturer C	White	1.75
PET-G 3		Manufacturer B	White	1.75

**Table 2:** Average emission rates (µg/h) of a given compound—ER and emission efficiency [µg/g] for ABS-based filaments.

Compounds	ABS 1		ABS 2		ABS 3	
	ER [µg/h]	Yield [µg/g]	ER [µg/h]	Yield [µg/g]	ER [µg/h]	Yield [µg/g]
formaldehyde	47.32 ± 3.38	5.78 ± 0.28	13.07 ± 0.61	1.51 ± 0.07	9.65 ± 0.68	1.12 ± 0.08
acetaldehyde	11.33 ± 0.63	1.38 ± 0.04	11.34 ± 0.32	1.31 ± 0.07	8.71 ± 0.52	1.01 ± 0.06
acetone	6.17 ± 0.27	0.75 ± 0.028	6.26 ± 0.18	0.72 ± 0.033	9.43 ± 0.75	1.09 ± 0.09
propionaldehyde	2.09 ± 0.07	0.26 ± 0.010	-	-	-	-
benzaldehyde	8.64 ± 0.48	1.06 ± 0.04	3.03 ± 0.15	0.35 ± 0.017	6.84 ± 0.41	0.79 ± 0.05
valeraldehyde	3.40 ± 0.16	0.41 ± 0.012	5.75 ± 0.27	0.66 ± 0.019	2.74 ± 0.19	0.32 ± 0.02
toluene	17.48 ± 0.69	2.14 ± 0.11	27.28 ± 1.06	3.15 ± 0.09	7.35 ± 0.58	0.85 ± 0.07
ethylbenzene	95.02 ± 6.12	11.61 ± 0.34	156.49 ± 4.65	18.04 ± 0.82	24.70 ± 1.72	2.86 ± 0.20
o-xylene	26.21 ± 1.09	3.20 ± 0.12	22.83 ± 0.75	2.63 ± 0.11	4.59 ± 0.28	0.53 ± 0.03
p-xylene	11.31 ± 0.54	1.38 ± 0.04	18.92 ± 0.83	2.18 ± 0.10	0.61 ± 0.04	0.07 ± 0.01
styrene	210.97 ± 6.65	25.79 ± 1.17	373.31 ± 14.93	43.04 ± 2.06	300.55 ± 15.06	34.80 ± 1.71
DMP	2.15 ± 0.08	0.26 ± 0.012	2.06 ± 0.11	0.24 ± 0.008	0.01 ± 0.00	0.00 ± 0.00
DEP	2.18 ± 0.09	0.27 ± 0.011	1.28 ± 0.05	0.15 ± 0.006	1.66 ± 0.12	0.19 ± 0.01
DBP	3.39 ± 0.11	0.41 ± 0.014	2.34 ± 0.08	0.27 ± 0.013	3.40 ± 0.24	0.39 ± 0.03
BBP	0.98 ± 0.048	0.12 ± 0.004	1.30 ± 0.04	0.15 ± 0.005	1.23 ± 0.07	0.14 ± 0.01
DEHP	0.40 ± 0.015	0.05 ± 0.002	0.47 ± 0.019	0.05 ± 0.002	0.57 ± 0.04	0.07 ± 0.01
DNOP	0.21 ± 0.011	0.03 ± 0.001	-	-	1.38 ± 0.08	0.16 ± 0.01

**Table 3:** Average emission rates (µg/h) of a given compound—ER and emission efficiency [µg/g] for PET-G-based filaments.

	PET-G-1		PET-G-2		PET-G-3	
Compounds	ER [µg/h]	Yield [µg/g]	ER [µg/h]	Yield [µg/g]	ER [µg/h]	Yield [µg/g]
Formaldehyde	11.17 ± 0.86	1.35 ± 0.08	5.39 ± 0.32	0.65 ± 0.03	6.76 ± 0.47	0.82 ± 0.05
Acetaldehyde	8.55 ± 0.61	1.03 ± 0.04	5.54 ± 0.38	0.67 ± 0.02	100.02 ± 4.20	12.14 ± 0.73
Acetone	3.27 ± 0.14	0.39 ± 0.02	2.44 ± 0.17	0.30 ± 0.01	2.79 ± 0.11	0.34 ± 0.02
Toluene	10.83 ± 0.93	1.31 ± 0.05	11.92 ± 0.79	1.45 ± 0.09	17.34 ± 1.25	2.10 ± 0.14
Ethylbenzene	-	-	2.00 ± 0.11	0.24 ± 0.01	2.13 ± 0.15	0.26 ± 0.02
o-Xylene	4.05 ± 0.29	0.48 ± 0.03	6.14 ± 0.31	0.75 ± 0.05	7.11 ± 0.52	0.86 ± 0.06
p-Xylene	1.89 ± 0.12	0.23 ± 0.01	2.47 ± 0.12	0.30 ± 0.02	1.45 ± 0.07	0.17 ± 0.01
DMP	0.05 ± 0.003	0.01 ± 0.001	4.13 ± 0.33	0.50 ± 0.04	2.34 ± 0.14	0.28 ± 0.02
DEP	1.23 ± 0.07	0.15 ± 0.01	3.27 ± 0.21	0.40 ± 0.03	8.35 ± 0.59	1.01 ± 0.07
DBP	1.87 ± 0.11	0.23 ± 0.02	3.54 ± 0.18	0.43 ± 0.03	8.08 ± 0.33	0.98 ± 0.04
BBP	1.07 ± 0.04	0.13 ± 0.01	4.57 ± 0.37	0.55 ± 0.04	25.72 ± 1.03	3.12 ± 0.21

**Table 4:** Parameters of particulate matter emissions in the 3D-printing process using ABS and PET-G-based filaments from three manufacturers.

Filament	Manufacturer	MAX PER	TP	Yield	ERp
		s^−1^	-	g^−1^	h^−1^
ABS	1	4.35 × 10^2^	5.00 × 10^5^ ± 3.03 × 10^3^	1.10 × 10^4^ ± 6.64 × 10^1^	1.67 × 10^5^ ± 1.01 × 10^3^
ABS	2	5.50 × 10^2^	6.49 × 10^4^ ± 7.14 × 10^2^	1.36 × 10^3^ ± 1.49 × 10^1^	2.16 × 10^4^ ± 2.38 × 10^2^
ABS	3	2.01 × 10^3^	1.11 × 10^6^ ± 6.22 × 10^3^	2.33 × 10^4^ ± 1.31 × 10^2^	3.69 × 10^5^ ± 2.07 × 10^3^
PET-G	1	1.80 × 10^2^	1.62 × 10^5^ ± 1.38 × 10^3^	3.56 × 10^3^ ± 3.04 × 10^1^	5.39 × 10^4^ ± 4.61 × 10^2^
PET-G	2	9.94 × 10^1^	1.51 × 10^5^ ± 9.80 × 10^2^	3.34 × 10^3^ ± 2.17 × 10^1^	5.04 × 10^4^ ± 3.27 × 10^2^
PET-G	3	1.64 × 10^2^	1.63 × 10^5^ ± 1.27 × 10^3^	3.51 × 10^3^ ± 2.74 × 10^1^	5.42 × 10^4^ ± 4.23 × 10^2^

**Table 5:** Average emission efficiency of gaseous products released from ABS- and PET-G-based filaments when heated at recommended operating temperatures.

Sample Name		Average Emission Efficiency, mg/g					
		CO_2_	CO	NO_2_	NH_3_	HCl	HCN
ABS	1	171 ± 32	17.8 ± 0.53	4.34 ± 0.43	0.04 ± 0.01	2.26 ± 0.48	5.61 ± 1.12
	2	83 ± 15	26.4 ± 1.1	0.89 ± 0.07	0	1.79 ± 0.07	3.44 ± 0.55
	3	71 ± 11	3.6 ± 0.03	2.67 ± 0.03	0.05 ± 0.01	0.55 ± 0.16	0.52 ± 0.08
PET-G	1	413 ± 74	35.8 ± 1.2	14.3 ± 1.3	0.37 ± 0.07	1.09 ± 0.22	1.06 ± 0.18
	2	507 ± 81	25.7 ± 1.1	2.42 ± 0.23	0.20 ± 0.03	4.14 ± 0.75	6.98 ± 1.14
	3	691 ± 110	17.4 ± 0.35	21.2 ± 1.78	0.44 ± 0.08	0.96 ± 0.16	0.31 ± 0.05

**Table 6:** Average emission efficiency of selected light hydrocarbons released from ABS- and PET-G-based filaments when heated at recommended operating temperatures.

Sample Name		Average Emission Efficiency, mg/g				
		CH_4_	C_2_H_6_	C_3_H_8_	C_2_H_4_	C_6_H_14_
ABS	1	0.50 ± 0.06	2.30 ± 0.27	3.16 ± 0.58	0.82 ± 0.14	2.03 ± 0.14
	2	0	3.14 ± 0.38	2.69 ± 0.47	0.71 ± 0.11	2.37 ± 0.23
	3	0	0	1.45 ± 0.16	0	0
PET-G	1	0	0	0.50 ± 0.10	0	0
	2	0.66 ± 0.06	3.92 ± 0.71	1.16 ± 0.23	0.89 ± 0.15	0.90 ± 0.09
	3	0	0	1.35 ± 0.24	0	0.30 ± 0.05

**Table 7:** Results of selected volatile carbon compounds during thermal degradation of ABS-based filaments at 255 °C and PET-G-based filaments at 245 °C for 60 min.

	Compound	CAS		ABS			PET-G	
			1	2	3	1	2	3
1	2-Butenal, 2-ethenyl-	20521-42-0	+	+	+			
2	Toluene	108-88-3	+	-	-	+	+	+
3	Cyclohexene, 4-ethenyl-	100-40-3	+	+	+			
4	Ethylbenzene	100-41-4	+	+	+	+	+	+
5	p-Xylene	106-42-3	+	+	+			
6	Styrene	100-42-5	+	+	+			
7	Benzene, (1-methylethyl)-	98-82-8	-	+	+			
8	Benzene, propyl-	103-65-1	-	+	+			
9	Benzaldehyde	100-52-7	+	+	+			
10	Acetophenone	98-86-2	+	+	+			
11	1-Dodecene	112-41-4	-	+	+			
12	Dodecane	112-40-3	-	+	+			
13	Benzenebutanenitrile	2046-18-6	+	-	+			
14	Tetradecane	629-59-4	-	+	+			
15	Dodecanal	112-54-9	-	+	+			
16	2,5-Cyclohexadiene-1,4-dione, 2,6-bis(1,1-dimethylethyl)-		+	+	+			
17	Hexadecane	544-76-3	-	+	-			
18	Benzene, 1,1′-(1,2-cyclobutanediyl)bis-, trans-	20071-09-4	+	+	+			
19	2-[1-(4-Cyano-1,2,3,4-tetrahydronaphthyl)] propanenitrile	57964-39-3	-	+	+			
20	3-[1-(4-Cyano-1,2,3,4-tetrahydronaphthyl)] propanenitrile	57964-40-6	-	+	+			

## Data Availability

The original contributions presented in the study are included in the article, further inquiries can be directed to the corresponding author.
